# Towards Inclusive Healthcare Delivery: Potentials and Challenges of Human-Centred Design in Health Innovation Processes to Increase Healthy Aging

**DOI:** 10.3390/ijerph17124551

**Published:** 2020-06-24

**Authors:** Catharina Thiel Sandholdt, Jason Cunningham, Rudi G.J. Westendorp, Maria Kristiansen

**Affiliations:** 1 Department of Public Health and Center for Healthy Aging, Faculty of Health and Medical Sciences, University of Copenhagen, Øster Farimagsgade 5, DK-1014 Copenhagen, Denmark; westendorp@sund.ku.dk (R.G.J.W.); makk@sund.ku.dk (M.K.); 2 Family Physician, Chief Executive Officer, West County Health Centers, 14045 Mill Street, P.O. Box 1449, Guerneville, CA 95446, USA; jcunningham@wchealth.org

**Keywords:** healthy aging, co-design, person-centered care, polypharmacy, health intervention, shared-decision making, multimorbidity, public health services, quality of life

## Abstract

National healthcare systems need to adjust services and operations to accommodate the needs of complex, aging populations living with multimorbidity and polypharmacy. This paper suggests the use of a human-centred design as a method to engage older adults and key professionals in innovation processes aiming to design person-centred healthcare services and improve quality of life in older adults. We outline three innovation phases and highlight how such processes can create engagement and new insights on how life experiences of older adult’s shape preferences, beliefs, and habits. It is important to incorporate these insights into the design of successful strategies for ensuring age-friendly healthcare services. Our viewpoint is contextualised through a small-scale case study focusing on polypharmacy in older adults. From this case study, we extracted three challenges to producing co-designed health research: recruitment, time and resources, and funding. We discuss how to address these challenges. We argue for the involvement of older adults and professional stakeholders at an early stage in the design process to align expectations and to increase the likelihood of successful implementation of healthcare innovations that improve the quality of life for older adults.

## 1. Introduction

Population aging poses a challenge for healthcare systems in terms of increased expenses and a rise in multimorbid patients with complex needs [1]. This shifting dynamic is creating an impetus to understand the needs of this new patient demographic and create effective and acceptable care, tailored to their unique needs, and to allow for as many healthy years as possible. This population, has a high prevalence of multimorbidity and requires care that is person-centred and coordinated, which is particularly challenging in the current healthcare environment [2]. Polypharmacy, a recognized health challenge in aging and multimorbid patients, is an example of an issue requiring a more person-centred care delivery model that provides more compassionate and effective care [3,4,5]. There is an appropriate shift towards a more person-centred care delivery model that provides more compassionate and effective care and improves the quality of life in older adults [6,7,8].

One essential aspect of delivering person-centred care is participation, specifically, collaboration and engagement [9]. In this commentary we elaborate on our experiences from research and practice, underlining the importance of involving the priorities and values of older adults in their care plan and building a structure for a person-centred healthcare system, which cultivates compassionate care [10]. We believe a co-design mindset is important when producing knowledge on person-centred care for older adults.

With the ambition to involve older adults and professionals stakeholders in the intervention development processes, human-centred design can be a relevant and reproducible method [11,12,13]. Methods from design research, such as human-centred design, are increasingly used in healthcare development, providing a framework for working with complex health problems and improving care trajectories by involving patients and health professionals in health innovation processes [14,15,16]. Engaging professional stakeholders and older adults in co-designed activities marks a shift from the more traditional scientific approaches of collecting data outside of the university, followed by analysis in objective surroundings [17]. Instead the human-centred design approach generates knowledge together with older adults and professional stakeholders, documenting their experienced challenges and formulating new hopes for the future [18,19]. The main aim when working with human-centred design is to generate empathy, to understand the older adult’s lived experience, biography, values, and priorities through participatory processes. In a healthcare system that wishes to emphasise patient ownership and develop future healthcare services centred around empathy, this mindset is highly relevant [20,21]. A person-centred healthcare delivery model that focuses on taking the preferences and values of the individual and his or her relatives into account when deciding on the provision of care, has major implications on the quality of life in older adults. Documenting a reproducible process for involving a more human-centred design approach to research is needed, including understanding the current challenges to adopting this novel approach on multiple levels. We therefore show the potentials of human-centred design, but also identify and discuss three main challenges for carrying out inclusive and co-designed health-intervention development: 1. Recruitment, 2. Time and resources and 3. Funding.

## 2. Materials and Methods

To contextualize this commentary, we elaborate on a recent innovation project to exemplify the steps and key challenges of using human-centred design in engaging older adults for the improvement of healthcare trajectories [22].

From 2018 to 2019, as part of a multinational European research collaboration on engaging narratives of older adults in healthcare delivery, the Copenhagen research team applied human-centred design as a method for exploring the factors contributing to polypharmacy [26,27]. The study was created as a case study for involving older adults and professional stakeholders in the research process. To produce knowledge on patient narratives and polypharmacy, we applied a range of methods for generating data through semi-structured interviews, field visits, field notes, and facilitation sessions [23,24,25,26,27]. The primary analytical method used was thematic analysis [28].

**Figure 1:**
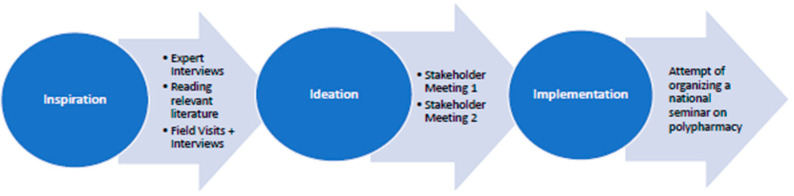
The three phases of innovation in human-centred design as carried out in the current case.

A core principle in human-centred design is to work iteratively through three phases of innovation: 1. Inspiration, 2. Ideation, and 3. Implementation, [29] (Figure 1). The main aim of this choice of method was to enable a democratic process, which could build understanding, empathy, and compassion for older adults.

### 2.1. Innovation Phase 1: Inspiration

In the inspiration phase, the goal is to create understanding for the defined problem, to challenge assumptions, and to begin to define the problem from the perspective of older adults. Design methods such as journey mapping, direct observation, shadowing, and expert interviews are applied [27]. The information generated from this phase is used to distil and develop insights that can offer clarity to the problem and generate ideas and opportunities for further research or investment within the Ideation phase. Empathy generated from direct observation, with a mindset of open exploration and understanding, is the key principle in the Inspiration phase, which can allow for a more compassionate and sustainable health intervention.

### 2.2. Innovation Phase 2: Ideation

In the Ideation phase, ideas are generated through a process of co-design with key stakeholders. Design methods such as “affinity clustering”, “how-might-we”, and “personas” are examples of methods used within this phase [27]. The goal of this phase is to further clarify the different aspects of the problem with key stakeholders and create specific ideas for collective action around identified opportunities for change.

### 2.3. Innovation Phase 3: Implementation

In the implementation phase, participants are guided through a process to create tangible solutions and to further test and refine assumptions. Prototyping is key during this phase of innovation. In the example of polypharmacy, products such as e-health tools or more policy-driven directives were identified as potential solutions.

## 3. Results

In the following section, we describe the steps taken and key challenges encountered during the three phases of innovation (Figure 1).

### 3.1. Inspiration Phase

We applied a two-fold strategy of engaging older adults and professionals on the topic of improving polypharmacy management. Firstly, we engaged experts in polypharmacy including professional stakeholders and researchers. Secondly, we interviewed five older adults living with polypharmacy and one general practitioner (GP) caring for older adults with multimorbidity and polypharmacy. Here, we encountered the first challenge: Recruitment. The eligibility requirements for participation were age 55 years and above, all genders, in active treatment for more than two chronic conditions, and taking more than five prescribed medications per day. We advertised for older adults with multimorbidity through communication channels of a large aging research centre, social media, and by the general population and civil society organisations engaged in the field of healthy aging. Snowballing was also used, resulting in contacts made with several people living with multimorbidity who were willing to participate in interviews following informed consent. The older adults who responded covered a broad range of socioeconomic groups, however, there was an overrepresentation of women living alone. The first author visited the participants for an interview. Whereas recruiting older adults proved not to be difficult, recruiting GPs was a challenge, with lack of time stated as the primary reason not to participate. A GP from a larger primary care clinic agreed to participate and the first author used shadowing as the method by following the GP for a working day. This required financial compensation, which needed to be allocated in the budget [25].

The inspiration phase was time-consuming, yet fruitful, as it enabled valuable insights of importance to be carried into the next phase. The multimorbid older adults showed us their everyday experiences of managing polypharmacy. They shared their frustrations of navigating in a siloed healthcare system with poor communication among doctors and they shared despondency over personal health information (and treatment requests) being “lost” in a system of information. The day spent shadowing a GP provided us with a broad overview of the busy workflow in the GP clinic, including specific knowledge about the different actors involved in prescribing, continuing, and de-prescribing medication.

### 3.2. Ideation Phase

Audio recordings from the visits in the patients’ homes were manually analysed using a thematic analytical approach. The field notes from the visit to the GP were contextualised by research literature on polypharmacy as well as the official guidelines and financial incentives affecting prescription practices. Based on this analysis, we arranged two ideation meetings with relevant professional stakeholders to discuss the information from the inspiration phase and to begin generating ideas for collective action around the topic of polypharmacy. The three professional stakeholders were the governmental institution responsible for administering healthcare, a non-governmental organisation (NGO) working with patient safety, and representation from the Danish digital health platform. The steps taken in the stakeholder meetings are listed in Table 1.

In the ideation phase we encountered the second challenge: time and resources. We were successful in bringing together relevant professional stakeholders, who were all influential in developing official health policies, for two meetings. Here, a strong sense of need for collective action and collaborative work arose from the co-design sessions and the intention to participate further was certainly present. The participants in the stakeholder meetings created a list of issues of concern to form a future collaboration based on prototyping and testing. The stakeholders identified the human-centred design methods applied through the inspiration and ideation phases as an important aspect leading to a shared commitment in further collective action. In spite of this positive and constructive experience, getting them committed for a more substantial process proved cumbersome due to structural and organisational barriers and lack of time and resources.

### 3.3. Implementation Phase

The challenge of time and resources made it difficult to pursue the third phase of innovation. A core group of the stakeholders continued collaboration with the aim of influencing official health-policy work through a national seminar on patient-centred treatment strategies within polypharmacy, based on the Scottish initiative called *Realistic Medicine* [30]. However, the lack of resources to allocate time and money for prototyping and testing made it impossible to continue to the implementation phase, illustrating some of the difficulties in carrying out co-designed health research. This highlights the third challenge: Funding. In order to design a participatory project based on human-centred design methods, funding must be allocated to ensure the ability of stakeholders to engage in the process throughout all three phases of innovation.

## 4. Discussion

Human-centred design offers tangible, reproducible, and scalable methods to make older adults and professional stakeholders a part of innovative healthcare processes. Further, the investment of time and effort to understand the experiences of older adults and engage key stakeholders should allow for more efficient and effective intervention development, dissemination, and adoption.

Inclusive healthcare delivery is of high importance when it comes to quality of life for older adults, essentially because it focuses on planning care trajectories that are tailored to the needs of the older adult and his or her life situation. This extends well beyond traditional types of health-related outcomes as it entails, for example, how to prescribe medicine for hypertension that enables the older adult to walk his or her beloved dog without worrying about having to use a toilet. A medicine choice based on knowledge of and discussion with the patient and close relatives will increase the chances of successful medicine compliance and thus reduce the risk of poor clinical outcomes and hospital admissions. This will allow for more older adults to experience a healthy and active life.

Through the use of a case study we have illustrated three challenges in human-centred design processes related to recruitment, time and resources, and funding. We found that the active involvement of older adults living with polypharmacy as a result of multimorbidity provided a contextual and multi-view perspective of the consequences that polypharmacy may have on everyday life and ways of improving care. These lived-life experiences form preferences, beliefs, and habits and are important to incorporate into any successful care strategy. However, the three identified challenges affected our ability to successfully carry out the final phase of innovation. In the following, we discuss each challenge and exemplify the barriers they can cause.

### 4.1. Recruitment

In order to execute a successful human-centred design process, understanding the real-world experience of the end-user, here being the older adult, is critical. We did not encounter challenges during recruitment for this case study, however, recruitment of socioeconomically disadvantaged older adults or ethnic minorities is often challenging and requires active outreach efforts [31,32]. Recruitment of health professionals and other stakeholders may be affected by lack of time or different organisational priorities resulting in a need for political negotiations and rearrangement of prioritisation to allow for stakeholders to commit to co-designed innovation projects.

A strategy for recruitment should therefore entail collaboration with relevant professional stakeholders such as municipal workers, patient associations, and the wide range of non-governmental associations relevant for recruiting and engaging participants.

### 4.2. Time and Resources

A strategy of allocation of time and resources are important to ensure a persistent and sustainable commitment to the project. When planning health innovation projects, it is important to remember that the process of facilitating and carrying through the three phases of innovation in human-centred design is a deliverance in itself; the bringing together of expertise, of building relationships, and of creating a space to rethink complex areas of concern. It is therefore important to allocate resources (economic, manpower, time) to the process itself. It is this care of the process from leadership courage and buy-in, to assignment of skilled staff, to time and resource allocation, that enables innovation to happen [11,33].

For further studies, it would be relevant to apply a strategy of involving older patients in all phases of the innovation cycle and have them more actively represented in activities such as stakeholder workshops. It would also be interesting to involve more varied patient groups and explore how design thinking can make the voices of marginalised patient groups part of (power) conversations and decision making. A large-scale study would also allocate resources for long-term measurement of the effects of such a process and explore how to meaningfully measure change(s) in care and empathy, which is a needed next step [34].

### 4.3. Funding

As already touched upon, well-distributed resources are vital. The majority of health research today is funded externally by private or public funds. When applying for grants, funders request detailed information on aims, methods, and expected outputs. These requirements fit poorly with human-centred design projects focusing on complex problems and iterative knowledge production. In a sustainable research set-up, the allocation of funds should be distributed to cover all aspects of involvement including, for example, prototype developments. A participatory innovation project holds many unknowns because the outcome is to be created together and expected project outcomes are thus difficult to formulate in advance. It further creates a need for investing significant time and energy in the early stages of formulating concrete research proposals or framework.

We argue that co-design methods, such as human-centred design, can be a valuable tool when doing research on complex issues, such as polypharmacy, due to the fact that it can produce knowledge on multiple levels. Everyday health practices, such as medicine consumption, as well as insights on systematic practices and procedures, such as prescribing and de-prescribing of medicine, can be evaluated and novel solutions can be identified as part of innovative research processes. The promotion of empathy through this methodological approach may furthermore enable kind and compassionate solutions in healthcare development.

Human-centred design methods are interesting and relevant ways of bridging perspectives of research and practice. They enable innovation processes by placing the perspectives of older adults at the centre and recognizing the value of interdisciplinary work [35,36]. Several examples of the growing trend of involving patients and their wishes in healthcare exist, [37,38,39,40] and we encourage this ongoing development. It is these examples and dialogues with funding institutions that can pave the way for a funding structure supporting participatory health innovation processes.

## 5. Conclusions

In conclusion, we argue for a need for adoption of reproducible research methods for involvement of older adults and key stakeholders to improve healthcare delivery for an aging population. Applying human-centred design methods in investigating the experienced consequences of diseases has proven to be a unique opportunity to explore an interdisciplinary approach and promote new ways of performing compassionate care with an aim to increase activity and health at older ages. However, it will require a shift towards a more open-ended process of stakeholder buy-in, topic selection, and prioritization. When initiating co-designed health innovation projects engaging older citizens, researchers should consider the three key challenges outlined here: recruitment, time and resources, and funding. We have formulated tentative strategies to address these and we encourage others to do the same with the hope of building a substantial body of shared knowledge.

## Figures and Tables

**Table 1:** Results from the meetings with professional stakeholders.

Method	Action	Result
Personas	The researchers presented their tentative findings from the inspiration phase using storytelling, pictures, and direct quotations to ground the information in the perspective of the older adults. Professional stakeholders took notes from what they heard. “What do you find: important/surprising/provoking/sad/constructive/uplifting by the persona-story presented?”	Creating empathy for the older adults. stakeholders could “put themselves” in the position of the multimorbid patients.
Affinity Clustering	The professional stakeholders presented their notes from the persona exercise to each other. Together they grouped the notes thematically, creating clusters of insight.	Each participant was given time for individual reflection and then participated in a collective process of insight generation, creating a shared common ground.
“How Might We?”	Stakeholders were asked to work in a collective ideation process in which they generated themes for collective action out of the insights from the brainstorming, by asking, “how might we?”.	Creating a bridge from diagnosing the problem to suggesting paths/areas of focus to pursue.
Visioning/Next Steps	The professional stakeholders engaged in a joint discussion and reflection on how to pursue the paths suggested in the “how might we” exercise.	A platform of shared responsibility was created, and an action plan was agreed upon.
Context-Specific Outcome of the Ideation Meetings		Specific themes were agreed upon and formulated by the professional stakeholders: 1. Development of a new professional role within the healthcare system that would have authority and responsibility for care-coordination and bridging the siloes of the healthcare system as it relates to polypharmacy (included a needed skill set of active listening, constructive curiosity, collaborative communication, and a willingness to embrace the inherent ambiguity in the delivery of care to multimorbid patients); 2. Patient knowledge as an asset to break down the existing medication prescribing power hierarchy; 3. Approaching medicine prescribing differently rather than by doing more (including allocating resources for reflection within prescribing and deprescribing, and using incentives to accommodate complexity and ambiguity); 4. Deprescribing guidelines for the multimorbid patients that incorporate multiple diseases, reflect diminishing return with increasing number of medications, and reflect the patient goals as a priority.
